# Comparison of Ultrasound and MRI with Intraoperative Findings in the Diagnosis of Peroneal Tendinopathy, Tears, and Subluxation

**DOI:** 10.3390/jcm13030740

**Published:** 2024-01-27

**Authors:** David M. Melville, Mihra S. Taljanovic, Lana H. Gimber, Matthew Miller, Aamir Ahmad, Dustin Sepich, L. Daniel Latt

**Affiliations:** 1 Department of Radiology, Mayo Clinic Arizona, Phoenix, AZ 85259, USA; melville.david@mayo.edu; 2 Departments of Medical Imaging and Orthopaedic Surgery, University of Arizona, Tucson, AZ 85719, USA; mihrat@radiology.arizona.edu; 3 Department of Radiology, University of New Mexico Health Sciences Center, Albuquerque, NM 87106, USA; 4 Kaiser Permanente Moanalua Medical Center, Honolulu, HI 96819, USA; lana.h.gimber@kp.org; 5 Department of Orthopaedic Surgery, University of Arizona, Tucson, AZ 85719, USA; mattmiller@arizona.edu; 6 Department of Orthopaedic Surgery, University of New Mexico Health Sciences Center, Albuquerque, NM 87106, USA; aahmad85@arizona.edu; 7 Banner Health, Peoria, AZ 85381, USA; dustin.sepich@bannerhealth.com

**Keywords:** peroneal tendonitis, peroneal tendon tear, peroneal tendon subluxation, ultrasound, MRI

## Abstract

Suspected peroneal tendinopathy, tears, and subluxation are often confirmed preoperatively using magnetic resonance imaging (MRI) or diagnostic ultrasound (US). No study has directly compared the accuracy of these tests for the diagnosis of peroneal tendon pathology. The purpose of this study is to directly compare MRI and US to intraoperative findings in patients who underwent surgery for suspected peroneal pathology to determine the imaging diagnostic accuracy. Operative records and diagnostic images for 21 consecutive patients who had both MRI and US prior to surgery for suspected peroneal tendinopathy, tears, or subluxation were retrospectively reviewed. The results of this review are compared with the intraoperative findings to yield the sensitivity and specificity for each imaging modality. For the diagnosis of peroneal tendon tears, US was found to have a sensitivity of 88% and specificity of 100%, compared to 100% sensitivity and specificity for MRI. In the diagnosis of peroneal tendinopathy, both US and MRI had a sensitivity and specificity of 100%. In diagnosing peroneal subluxation, US was 100% sensitive compared to 66% for MRI, and both were 100% specific. In conclusion, US was found to be more effective in diagnosing peroneal subluxation and MRI was slightly more accurate in the diagnosis of peroneal tendon tears.

## 1. Introduction

Peroneal tendon disorders are a common cause of chronic lateral ankle pain [1], but are commonly missed initially [2]. Patients typically present with chronic lateral ankle or hindfoot pain, frequently localized to the retromalleolar or inframaleollar region that worsens with activity, occasionally accompanied by a clicking or popping sensation [3]. Potential diagnoses include peroneal tendinopathy (tendinosis/tenosynovitis), peroneal tendon tear (including longitudinal split tears), and peroneal tendon instability (subluxation/dislocation) [4]. These disorders tend to occur concurrently and are associated with traumatic injuries, frequently related to ankle inversion, as well as athletic pursuits with repetitive microtrauma along with chronic diseases, such as diabetes mellitus, inflammatory arthropathies, and metabolic arthropathies, including crystal deposition diseases [5]. Up to 30% of patients undergoing surgery for ankle instability have a peroneal tendon tear, but both tendons are rarely torn simultaneously [5,6]. Spontaneous tendon rupture typically occurs in the setting of pre-existing tendinopathy [7].

Peroneal tendon injuries with instability include anterior subluxation and dislocation of the peroneal tendon(s) from the retromalleolar groove due to superior peroneal retinacular (SPR) injuries, which may be mistaken for a lateral ankle sprain when occurring in isolation [8]. SPR injuries usually result from the sudden contraction of peroneal muscles during an acute inversion of a dorsiflexed ankle or during the forced dorsiflexion of an everted foot [9]. In this setting, subluxation is typically transient and recurrent [5]. Instability may also be manifested in the presence of an intact SPR when the peroneus brevis and longus tendons sublux over one another in the retromalleolar groove [10]. Due to transient nature of this subluxation, it is difficult to detect using static imaging [11].

Unfortunately, all of these disorders are often difficult to differentiate clinically, thus necessitating the use of advanced imaging with magnetic resonance imaging (MRI) or ultrasound (US) evaluation [5,12,13]. The choice between these imaging modalities is usually based on clinician preference, as definitive evidence for the superiority of one over the other is lacking. The choice of modality may also be affected by implanted devices, the availability of local equipment, and expertise [5]. The increasing availability and portability of and familiarity with musculoskeletal US also impact this choice, as many clinicians favor expedience in reaching a diagnosis and moving quickly to definitive surgical treatment. 

A few studies have quantified the sensitivity and specificity of MRI [14,15,16,17] or US [18,19,20] in the evaluation of peroneal tendon injuries using intraoperative evaluation as the gold standard. Two studies compared US to intraoperative findings and found US to have a sensitivity of 100% and specificity of 88–90% in the detection of peroneal tendon tears [19,20]. Another study comparing MRI and intraoperative findings showed MRI to be 44% sensitive and 99% specific in detecting tears of the peroneus brevis and 50% sensitive and 99% specific for peroneus longus tears [17]. Other studies have reported the sensitivity and specificity of US to make these diagnoses as 100% and 85%, respectively [18]. The literature shows that MRI is useful in identifying the presence of longitudinal split tears of the peroneal tendons, but does not reliably predict the degree of peroneal tendon pathology, and often underestimates the extent of peroneus longus tears [21]. Conversely, US can be performed as a dynamic examination that may improve its ability to detect tendon instability, including intra-sheath subluxation [22]. The purpose of the present study is to compare MRI and US against intraoperative findings to determine which imaging modality is the best suited for diagnosing peroneal tendon injuries.

## 2. Materials and Methods

After institutional review board approval, the medical records and diagnostic images of 21 patients with lateral ankle pain who had undergone surgery over a seven-year period by a single surgeon for the treatment of suspected peroneal tendinopathy, tears, or subluxation or dislocation, including intra-sheath subluxation, were retrospectively reviewed. Patients were included if they: (1) had retromalleolar or inframalleolar lateral ankle pain, (2) had undergone both MRI and US examination, (3) the results of the MRI and US confirmed the presence of peroneal tendon pathology, and (4) had undergone surgery for peroneal tendon exploration with possible repair. The patient population consisted of 8 males and 13 females, with an average age of 41 years (range of 15–71 years). Prior to surgery, each patient had undergone a trial of conservative treatment, including some combination of anti-inflammatory medication, bracing, and physical therapy. After failing to achieve symptomatic relief, they had been sent for MRI and US to confirm the diagnosis of peroneal tendon injury, which confirmed the presence of peroneal tendon tear or instability and led to the indication for surgery. 

### 2.1. Ultrasound 

The US examination of the peroneal tendons was performed with the patient in the supine position, the knee joint was semi-flexed, and the ankle was internally rotated. A high-resolution 8–18 MHz linear hockey stick transducer with a small footprint was used for grayscale and color/power Doppler imaging. To optimally evaluate these structures and avoid tendon displacement due to excess transducer pressure, generous amounts of US gel were applied to image along the curved surface of the lateral malleolus. The examination consisted of: (1) Axial/short-axis images obtained perpendicularly to the long axis of both tendons, with scanning from proximal to distal sites, and included the distal portion of the peroneal musculature, the myotendinous junction, and both tendons to their distal insertion sites. The tendons were examined together until they split at the level of the peroneal tubercle at the lateral aspect of the calcaneus, after which each one was examined separately. A similar examination was then performed in the longitudinal axis. Additionally, the tendon sheath was assessed for thickening, the size of the synovial fluid complex, and hypervascularity during color or power Doppler investigation. (2) A dynamic examination of the peroneal tendons in the retromalleolar groove evaluated for tendon subluxation/dislocation associated with SPR injuries or intra-sheath subluxation. These short-axis images were obtained in the supine position during resisted ankle dorsiflexion–eversion. (3) The patient was subsequently turned to a prone position for an improved evaluation of the plantar aspect of the peroneus longus tendon in both the long and short axes as it coursed obliquely from its distal insertion at the plantar aspect of the first tarsometatarsal joint to the cuboid tunnel and the lateral aspect of the calcaneus. Throughout the US evaluation, panoramic imaging and tissue harmonics were employed to better depict the pathology, as needed.

### 2.2. MRI

MR imaging of the ankle was performed using a dedicated transmit–receive multichannel extremity coil with the patient in the supine position and the ankle joint in neutral position with approximately 20 degrees of plantar flexion on a 1.5T or 3T MR imaging platform [5]. Twenty degrees of plantar flexion were employed to enhance the separation of the peroneal tendons within the common peroneal sheath in the axial plane. The imaging protocol consisted of fast spin-echo T1-weighted sequences in the axial, coronal, and sagittal planes, sagittal short tau inversion-recovery (STIR) sequence, axial T2-weighted sequence with fat saturation, and coronal proton-density-weighted sequence with fat saturation. This three-plane examination permits the adequate evaluation of the peroneal tendons and their surrounding structures. When pathology was suspected along the plantar aspect of the peroneus longus tendon, additional MRI sequences were obtained with the long axis along the metatarsal bones. 

### 2.3. Intraoperative

**Figure 1:**
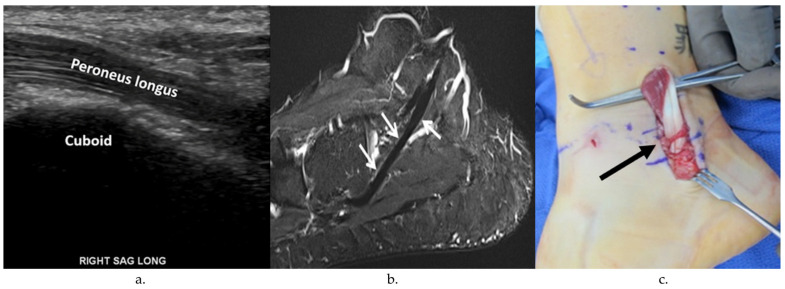
US, MRI, and intraoperative photos of peroneal tendinosis. (**a**) Long-axis (longitudinal) US image along the peroneus longus tendon at the lateral aspect of the calcaneus and along the cuboid groove shows hypoechogenicity of the tendon fibers consistent with tendinosis. (**b**) Sagittal STIR MR image of the same ankle shows mild thickening with scattered subtle intrinsic intermediate signal in the peroneus longus tendon consistent with tendinosis. (**c**) Intraoperative image of the lateral aspect of the left ankle showing significant tendinopathy of the peroneus longus (black arrow).

**Figure 2:**
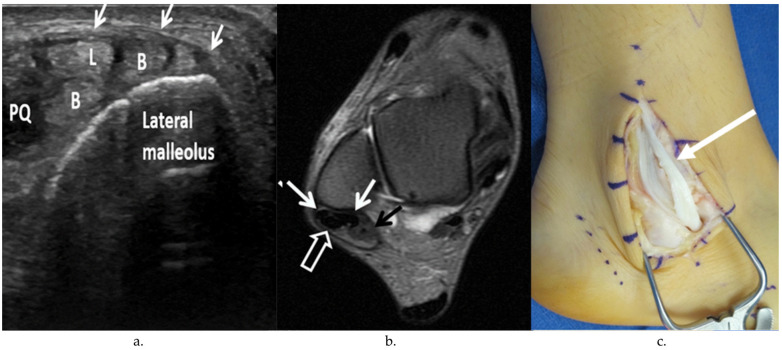
US, MRI, and intraoperative photos of peroneal tendon tears. (**a**) Transverse (short axis) grayscale US image of the right ankle in the retromalleolar region shows a longitudinal split tear of the peroneus brevis tendon with 2 hemi-tendons (B), intact peroneus longus tendon (L), and accessory peroneus quartus (PQ) under the superior peroneal retinaculum (SPR) (arrows). (**b**) Axial T2-weighted MR image with fat saturation of the right ankle shows a longitudinal split tear of the peroneus brevis tendon (white arrows) in the retromalleolar region. The peroneus longus tendon (open arrow) is intact. Note peroneus quartus at the posteromedial aspect of the peroneal tendons (black arrow). (**c**) Intraoperative image of the lateral aspect of the right ankle showing a longitudinal split tear of the peroneus brevis (black arrow).

**Figure 3:**
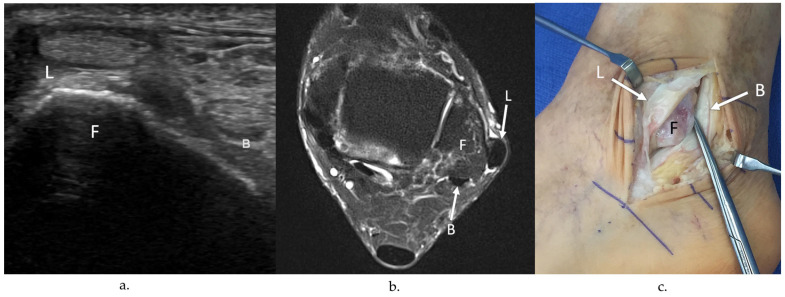
(**a**) Transverse (short axis) grayscale US image of the left ankle shows anterior dislocation of the peroneus longus tendon (L) from the lateral retromalleolar groove (F), secondary to a SPR tear. The peroneus brevis tendon (B) is normally located in the retromalleolar groove. (**b**) Axial T2-weighted MR image with fat saturation of the same ankle confirms anterior dislocation of the peroneus longus tendon (L) from the retromalleolar groove of the fibula (F) with normally located PB tendon (B). (**c**) Intraoperative image confirms anterior dislocation of the peroneus longus (L) tendon, secondary to a SPR tear. Note the normal retromalleolar location of the peroneus brevis tendon (B).

Peroneal tendon exploration and possible repair were performed in the lateral decubitus position. A longitudinal incision was made either along the course of the peroneal tendons or overlying the distal fibula, depending on whether lateral ankle ligament reconstruction was to be concomitantly performed. The superior and inferior peroneal retinacula were opened sharply, and the peroneal tendons were dislocated from the retromalleolar groove and were inspected. Peroneal tendinopathy was identified by the presence of a hypertrophic or erythematous tenosynovium or through the degeneration of the tendon substance with loss of collagen fibrils (Figure 1). The length and location of peroneal tendon tears was documented photographically with a ruler held up to the tendon (Figure 2). The presence of peroneal tendon subluxation was determined by the inspection of the attachment of the fibrocartilaginous ridge and SPR to the lateral extent of the fibular groove (Figure 3).

### 2.4. Imaging Review

Following surgery, the MR images were systematically re-evaluated by two fellowship-trained musculoskeletal radiologists specifically for the presence of tendinopathy, tears, and subluxation or dislocation of the peroneus longus and brevis tendons. The radiologists were blinded to the previous reading of the images and to the intraoperative findings. During the reading of the MRIs, peroneal tendinosis was determined to be present when there was tendon thickening with increased intra-substance signal. Similarly, tendon thickening with hypoechogenicity indicated tendinosis at US. Tenosynovitis was determined by the presence of more than 3 mm of fluid surrounding the tendons, as well as tendon sheath hyperemia on color/power Doppler US images. Longitudinal split tears were determined to be present when the axial/transverse images demonstrated the presence of two hemi-tendons. Partial-thickness tears were deemed present when the tendons were either thinned or thickened with an irregular intermediate or hyper-intense signal along the tendon surface on MRI. Partial-thickness tears at US were diagnosed by the presence of a focal hypoechoic cleft along with tendon thickening or attenuation. Complete tears at US and MRI were identified by the full-thickness disruption of tendon fibers with or without interposed fluid and/or tendon retraction. Extra-sheath peroneal subluxation or dislocation was present when one or both peroneal tendons were located anteriorly to the retromalleolar groove in the presence of a tear of the SPR. Intra-sheath subluxation was defined as abnormal tendon position on static MR images or the swapping of peroneal tendon positions within the lateral retromalleolar groove during dynamic US evaluation. The US images, including cine loops, were re-evaluated and subsequently compared with the original reports. The original US studies were reported by one of four fellowship-trained MSK radiologists, and the consensus re-evaluation by two blinded readers did not reveal any disagreements with the initial US imaging interpretation. The reported findings were scored and tabulated. Consensus grading was used to assign a score of 0 (no pathology present), 1 (possible pathology present), or 2 (definite pathology present) for the presence of tendinopathy, tears, and subluxation or dislocation in both MRI and US.

### 2.5. Data Analysis

The tabulated US and MRI findings were then compared to the intraoperative findings. For this analysis, the imaging finding was considered positive when it was either possible (score = 1) or definitive (score = 2). Sensitivity and specificity for each pathologic condition were calculated for both MRI and US. Sensitivity was calculated as the ratio of the number of findings detected by imaging to those confirmed at surgery (true positive/(true positive + false negative)). Specificity was calculated as the ratio of the number of findings correctly ruled out by imaging to those found to be absent at surgery (true negative/(true negative + false positive)). 

## 3. Results

Thirteen of twenty-one patients had peroneus brevis tendon longitudinal split tears and three had partial-thickness tears. Two of twenty-one patients had peroneus longus tendon tears, one complete rupture, and one longitudinal split tear (Table 1). The MRI results were concordant with the surgical findings (100% sensitivity and 100% specificity) for all peroneal tendon tears. US failed to diagnose two PB longitudinal split tears (85% sensitivity and 100% specificity). Eighteen of twenty-one patients had tendinopathy and three intra-sheath subluxations of the peroneal tendons. US was found to be superior to MRI for the diagnosis of subluxation with 100% sensitivity and 100% specificity, compared to 66% sensitivity and 100% specificity for MRI (Table 1). There was a single peroneus longus tendon dislocation with 100% concordance between MRI and US.

## 4. Discussion

The goal of this study was to compare US and MRI with intraoperative findings for the diagnosis of peroneal tendinopathy, tendon tears, and tendon subluxation or dislocation, including intra-sheath subluxation. We found that neither imaging modality was clearly superior, with each having specific strengths and weaknesses. US was found to be more sensitive than MRI for the detection of intra-sheath peroneal subluxation, but somewhat less sensitive in the detection of peroneus brevis tendon tears and equally sensitive in the detection of other peroneal tendon pathologies. 

### 4.1. Peroneus Brevis Tear

Peroneus brevis tendon tears are a common cause of lateral ankle pain with longitudinal split tears occurring most frequently [5,23]. While these types of tears may be seen in any age group, they often result from degeneration or repetitive microtrauma with subsequent tendon thinning, which progresses to a tear propagating from the level of the retromalleolar groove [24]. Less commonly, peroneus brevis tears result from acute athletic injuries and contribute to chronic lateral ankle instability [25,26]. Given the relatively high rate of these tears in patients with lateral ankle pain, it is particularly important to assess the diagnostic accuracy of US and MRI in these cases. 

The present study found MRI to have 100% sensitivity and specificity in detecting peroneus brevis tears. Previous studies showed MRI to have sensitivities in the range of 44–56% and specificities in the range of 50–99% in detecting peroneus brevis tears (Table 2) [14,16,17,24]. Our findings may have differed from those of prior studies for several reasons. The first is that peroneus brevis tears were very common in this series, leading to a very high probability of finding a tear at surgery. A second factor is that the present study utilized two fellowship-trained musculoskeletal radiologists to read the MR images, which may have decreased the possibility of missing tears. A final factor is that we considered a score of both 1 (probably torn) and 2 (definitely torn) as a positive read. If we considered only scores of 2 as torn, the sensitivity would have dropped to 86% and the specificity to 88%.

In comparison, we found US to have 85% sensitivity and 100% specificity in diagnosing peroneus brevis tears. Previous studies have found sensitivities of 100% and specificities of 90% [18], and another study found a sensitivity of 100% and a specificity of 100% (Table 3) [27]. There are a few possible explanations for the slightly decreased accuracy of the present study to diagnose peroneus brevis tears. False positives (positive on imaging, but negative on surgical evaluation) may have occurred due to the healing of the tear that occurred between the time of imaging and surgery. Moreover, false negatives (negative on imaging, but positive at surgery) can also occur due to partial healing if it prevents the two halves of the split tendon from separating from each other. This could explain why peroneus brevis tendon tears are somewhat better evaluated using MRI. Furthermore, MRI affords a better visualization of predisposing osseous anatomy, such as a prominent peroneal tubercle or convex fibular groove, or the presence of associated lateral malleolar bone marrow edema [28,29], while predisposing anatomic variants, such as a low-lying peroneus brevis or a peroneus quartus, may be seen at both MRI and US [28,30].

### 4.2. Peroneus Longus Tears

Peroneus longus tendon tears occur less commonly than brevis tendon tears due to the anatomy within the peroneal tunnel; however, peroneus longus tendon tears at the level of the retromalleolar groove are often associated with peroneus brevis tendon tears [28,31]. Isolated peroneus longus tendon tears occur more commonly in the midfoot at the level of the cuboid bone due to increased friction or direct trauma [30]. A hypertrophied peroneal tubercle or fracture of the os peroneum may be seen with peroneus longus tendon tears [17,30,32]. Previous studies have found MRI has 50% sensitivity and 96% specificity in diagnosing tears of the peroneus longus [17]. Another study found MRI to be 67% sensitive and 90% specific in identifying partial peroneus longus tendon tears, and 16.7% sensitive and 91.9% specific in diagnosing longitudinal split tears of the peroneus longus tendon [24]. In a prospective study of ankle tendon tears, US was found to be 75% sensitive and 100% specific with just three surgically proven peroneus longus tendon tears [20]. In our study, both US and MRI were shown to be effective at imaging peroneus longus tendon tears, with a 100% sensitivity and 96% specificity in both modalities; however, the similarly small number of peroneus longus tendon tears in this series makes a definitive conclusion difficult. 

### 4.3. Peroneal Subluxation/Dislocation

Recurrent peroneal instability is defined as an abnormal position of the peroneal tendons at the level of the lateral malleolus and consists of two distinct types [11]. The first and most common is pre-fibular anterior dislocation–subluxation, which most frequently results from a stripping or avulsive injury to the SPR at its fibular attachment [11,27]. This results in the anterior subluxation or dislocation of the peroneal tendons out of the retromalleolar groove. Chronic tendon instability predisposes to additional peroneal tendon pathology, as well as osseous remodeling of the subjacent lateral fibular cortex [30]. The second type of instability is retro-fibular/retromalleolar intra-sheath subluxation, which occurs in the absence of SPR injury or other trauma and consists of abnormal motion of the peroneal tendons relative to one another within the retromalleolar groove [10].

US is widely regarded as the best imaging modality to detect both intra-sheath subluxation or extra-sheath anterior subluxation or dislocation of the peroneal tendons [11,12,22,27,33,34]. The present study confirmed the effectiveness of US in detecting peroneal tendon subluxation or dislocation (sensitivity 100%) and found it to be far superior to MRI, which was found to have a 66% sensitivity in this series and failed to detect one of three intra-sheath, or transient anterior, subluxations. The difference in sensitivity likely resulted from the static nature of MRI, as the subluxating tendons were located within the peroneal groove with normal positioning at rest during the MRI acquisition, whereas during dynamic US evaluation, the examiner can have the patient actively dorsiflex and evert the ankle, which allows the peroneal tendons to switch their positioning within the retromalleolar groove or drive them out of the groove to identify the presence of intra-sheath subluxation or anterior/pre-fibular subluxation or dislocation. This makes US useful in confirming physical examination findings in these cases while confirming the presence of concomitant tendon pathology. On the other hand, with retro-fibular/retromalleolar intra-sheath subluxation, the peroneal tendons do not displace over the lateral malleolus and imaging is required for diagnosis [10].

### 4.4. Limitations

There are several limitations to this study. First, the use of intraoperative findings as the gold standard limited the study population in two ways: (1) including only patients who underwent surgery excluded a large number of patients who had tendon pathology at imaging but responded to non-operative treatment, and (2) patients who had retromalleolar pain but negative peroneal tendon imaging were not taken to surgery unless there was some other reason to do so (e.g., lateral ankle ligament injury), potentially leading to the exclusion of patients who may have had peroneal tendon pathology and falsely negative imaging. 

Second, the method by which the imaging was reviewed was another source of potential bias. While the two MSK radiologists reviewing the MRI examinations were blinded to the intraoperative findings and initial image interpretation, the study lacked normal peroneal tendon controls. Moreover, the use of single-session consensus grading by the two reviewing radiologists limited the measure of both intra- and inter-observer reliabilities. Having three musculoskeletal radiologists performing multiple readings separated in time would have provided more information on the reliability of interpretation. 

Finally, looking to future studies, MSK radiologists could interpret the available US images at separate time points with and without the corresponding MRI examination. Emerging US advances should be incorporated into further research efforts, including the use of shear-wave elastography [35]. Recent evidence suggests pathological tendons may have reduced shear-wave elastography velocity compared to the healthy controls, and future prospective studies comparing US and MRI for the evaluation of peroneal tendon pathology should include elastography [36]. Contrast-enhanced US (CEUS) has been recognized as a diagnostic tool for tendinopathy and the assessment of surgical outcomes, particularly the rotator cuff and Achilles tendon [37]. Future studies could evaluate the utility of both pre- and post-operative peroneal tendon CEUS. Three-dimensional US has shown promise in evaluating the morphology of rotator cuff tears [38], and 3D US may also increase the sensitivity of US to diagnose peroneus brevis tears by better demonstrating the tendon morphology in the setting of partially healed or developing split tears. 

## 5. Conclusions

The principal finding of this study was that both US and MRI were effective in diagnosing peroneal tendon injuries. The study found US to be more effective in diagnosing tendinopathy and subluxation and MRI to be slightly more effective in diagnosing tears. Future research could prospectively follow a cohort of patients with clinical signs and symptoms of peroneal tendon disorders (rather than only those who underwent surgery) to determine the positive predictive and negative predictive values of both imaging modalities. Such a prospective study could also evaluate the effectiveness and appropriate timing of surgical intervention in the treatment of patients with peroneal tendon disorders. 

## Figures and Tables

**Table 1:** Peroneal tendon pathology identified intraoperatively with comparison of MRI and US results.

Pathology	Intraoperative Findings	MRI (Sensitivity/Specificity)	US (Sensitivity/Specificity)
Peroneus brevis tear	16	100/100%	88/100%
Peroneus longus tear	2	100/100%	100/100%
Tendinopathy	18	100/100%	100/100%
Tendon subluxation	3	66/100%	100/100%

**Table 2:** Comparison of studies of MRI evaluations of PB tears.

Study	Sensitivity	Specificity
Present	100%	100%
Lamm [14]	83%	75%
Khoury [16]	91% *	50% *
Park [17]	44%	99%
Sharpe [24]	62.9% **	93.3% **

* Percentage not reported, calculated from data provided. ** Reported values for peroneus brevis longitudinal split tears.

**Table 3:** Comparison of studies of US evaluations of PB tears.

	Sensitivity	Specificity
Present Study	85	100
Grant [18]	100	90
Neustatder [27]	100	100

## Data Availability

Datasets presented in this article are available only on request from the corresponding author due to patient privacy restrictions.
